# A Novel Peel to Prevent Post‐Inflammatory Hyperpigmentation After CO_2_ Resurfacing for Acne Scars

**DOI:** 10.1111/jocd.70366

**Published:** 2025-07-30

**Authors:** Xiaozhun Hang, Davin Sui Lim

**Affiliations:** ^1^ Queensland Institute of Medical Research Berghofer Herston Queensland Australia; ^2^ Cutis Clinic Indooroopilly Queensland Australia

**Keywords:** acne scars, chemical peel, CO_2_ laser, Fitzpatrick skin types, laser resurfacing, pigmentation prevention, post‐inflammatory hyperpigmentation

## Abstract

**Background:**

Atrophic acne scars are a common dermatological concern, often requiring fractional carbon dioxide (CO_2_) laser resurfacing for effective treatment. However, in darker skin types (Fitzpatrick III–V), the high risk of post‐inflammatory hyperpigmentation (PIH) remains a significant limitation, affecting treatment outcomes.

**Aims:**

This study evaluates the efficacy of a novel pre‐ and post‐treatment peel in reducing PIH following fractional CO_2_ laser resurfacing, particularly in higher Fitzpatrick skin types. The primary outcome was the incidence and severity of PIH, while secondary outcomes included healing time and changes in acne scar grading.

**Method:**

Twenty‐nine patients underwent fractional CO_2_ laser resurfacing, with the study group (*n* = 15) receiving adjunctive peeling treatment before and after the procedure. PIH severity was assessed using the Post‐Inflammatory Hyperpigmentation Area and Severity Index (PIHASI) at Week 6. Healing time and changes in acne scar grading (Goodman and Baron scale) were also analyzed.

**Results:**

The peel‐treated group had a lower median PIHASI score (0.0 [IQR: 0.0–0.2]) than the no‐peel group (0.2 [IQR: 0.2–0.4]) at Week 6. The Mann–Whitney *U* test showed a statistically significant difference (*z* = 2.427, *p* = 0.015, exact *p* = 0.016). Re‐epithelialization time was comparable (Day 6.4 in peel group vs. Day 6.2 in control group), and no significant difference was observed in acne scar improvement (scar reduction: 1.27 ± 0.59 vs. 1.36 ± 0.63, *p* = 0.694). No adverse events were reported.

**Conclusion:**

The novel peel effectively reduces PIH risk following fractional CO_2_ laser resurfacing without affecting scar outcomes. While it offers a promising approach for Fitzpatrick III–V patients, further studies with larger cohorts are needed to validate its long‐term efficacy.

## 1 Introduction

Acne scars, particularly atrophic scars, are a common dermatological concern, often resulting in significant textural irregularities and psychosocial distress [1]. Jacob's classification divides atrophic acne scars into ice pick, boxcar, and rolling scars, each requiring targeted approaches for optimal correction [2]. Among resurfacing modalities, fractional carbon dioxide (CO_2_) laser remains the gold standard for dermal remodeling. It effectively treats all three types of atrophic acne scars by inducing collagen remodeling, neocollagenesis, and fibroblast activation, with studies demonstrating its superiority over other resurfacing modalities [3].

A major limitation of ablative CO_2_ laser resurfacing, particularly in darker skin types (Fitzpatrick III–VI), is the risk of post‐inflammatory hyperpigmentation (PIH) [4]. This occurs due to prolonged melanocyte stimulation and increased epidermal cytokine activity from inflammatory damage to basal keratinocytes, leading to dyschromia and potentially compromising treatment outcomes [5]. Up to 92% of patients with Fitzpatrick skin type IV and higher develop PIH following ablative CO_2_ laser treatment [6]. Given this high incidence, PIH represents the rate‐limiting factor in the use of CO_2_ laser for atrophic acne scars, particularly in higher Fitzpatrick skin types (FST).

To mitigate PIH risk, various approaches have been explored. Non‐ablative fractional lasers (NAFL) and radiofrequency microneedling (RFM) have gained popularity due to their lower PIH risk [7, 8]. However, these modalities do not match the efficacy of CO_2_ laser in dermal remodeling [9]. Within ablative laser resurfacing, fractional CO_2_ lasers offer advantages over fully ablative CO_2_ lasers, as fractional technology minimizes epidermal disruption while still inducing deep collagen remodeling [9]. Additional modifications to laser density, lasing patterns, pulse duration, and epidermal cooling have been employed to reduce heat accumulation and lower the risk of PIH [10].

Once established, PIH can persist for several years, significantly impacting patient satisfaction and requiring prolonged intervention [11]. A wide range of topical, systemic, and procedural treatments exist for PIH, but prevention remains the primary objective (Table 1). Strategies for PIH prevention in CO_2_ laser resurfacing include topical tyrosinase inhibitors, corticosteroids, short‐pulsed lasers, photoprotection, and platelet‐rich plasma (PRP) [5, 10, 12, 13, 14, 15, 17, 19]. The use of post‐procedure agents such as topical fusidic acid has also been explored in clinical studies [16]. However, an optimal solution balancing effective scar remodeling with minimal PIH risk remains an unmet clinical need.

**TABLE 1:** Frequently adopted modalities to reduce the incidence of PIH following CO_2_ ablative laser.

Treatment modality	Mechanism of action
Photoprotection measures with sunscreen [12]	Reduces UV‐induced melanogenesis and prevents worsening of PIH
Cooling (pre‐, intra‐, and post‐CO_2_ lasing) [10]	Minimizes thermal injury, reducing melanocyte stimulation
Laser variables [13] Short pulse duration Low density Spot randomization	Optimizes laser parameters to minimize heat accumulation Reduces thermal diffusion, limiting melanocyte activation Minimizes overlap, reducing cumulative heat exposure Prevents localized heating, ensuring even energy distribution
Topical tyrosinase inhibitors (hydroquinone) [14]	Reduces melanin production by inhibiting tyrosinase
Topical corticosteroids [5, 15]	Suppress inflammatory cytokines, reducing melanocyte stimulation and mitigating PIH risk
Topical fusidic acid [16]	Reduces postoperative inflammation through anti‐inflammatory properties (inhibiting proinflammatory cytokines of TNF‐α, IL‐1β, and COX‐2)
Tranexamic acid [17]	Modulates the plasminogen pathway to decrease melanogenesis
Antioxidants such as 1% kojic acid, 5% niacinamide [18]	Reduces oxidative stress on melanocytes, preventing excess pigmentation
Platelet‐rich plasma (PRP) [19]	Activates the extracellular signal‐regulated kinase (ERK) pathway while inhibiting TGF‐β1, reducing melanin synthesis Promotes basement membrane repair through platelet degranulation and the release of laminin, collagen IV, and tenascin
Neurotoxin (Botulinum toxin‐A) [20]	Suppresses epidermal melanogenesis by decreasing tyrosinase activity and reducing melanin production
Polynucleotides [21]	Decrease melanin content, tyrosinase activity, and MITF/TRP‐1 expression, Activate ERK and AKT pathways, leading to suppressed melanogenesis
Exosomes [22]	Regulates homeostasis of melanogenesis Reduce intracellular melanin levels

Here, we explore a novel peel for use in both pre‐ and post‐treatment to assess its efficacy in reducing PIH following CO_2_ laser resurfacing, particularly in patients with darker FST.

## 2 Materials and Methods

### 2.1 Study Design

This was a prospective, single‐center, randomized, controlled, evaluator‐blinded study conducted in accordance with the Declaration of Helsinki. Informed consent was obtained from all participants prior to study enrollment. The study was supervised by a Fellow of the Australasian College of Dermatologists (FACD) and conducted by a team comprising a cosmetic physician and dermatology nurses.

### 2.2 Study Objective

The primary objective was to compare the incidence of PIH in the treatment group receiving a MelanoPro Peel System (Dermalogica Pro, Carson, CA, USA) versus the control group receiving no peel.

### 2.3 Patient Recruitment and Randomization (Figure 1)

**FIGURE 1:**
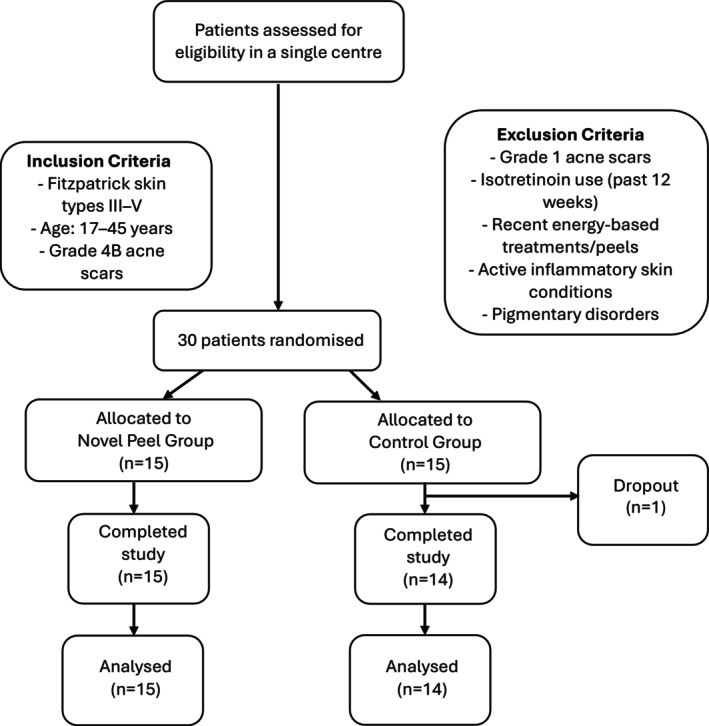
Flow chart: patient recruitment, randomization and analysis.

A total of 30 patients were recruited and randomized into two groups:

- Treatment group (*n* = 15): Received the MelanoPro Peel System
- Control group (*n* = 14): Received no peel. One patient dropped out, resulting in a final sample size of 14 patients in the control group.

### 2.4 Inclusion Criteria

- FST III–V
- Age range: 17–45 years
- Grade 4B acne scars (as per the Goodman and Baron scale)

### 2.5 Exclusion Criteria

- Patients with Grade 1 acne scars (macular erythematous scars or PIH)
- Use of isotretinoin within 12 weeks prior to study enrollment
- History of energy‐based device treatments or chemical peels within 12 weeks prior to the study
- Presence of inflammatory skin conditions, including active acne, which could confound the Post‐Inflammatory Hyperpigmentation Area and Severity Index (PIHASI) scores
- Patients with pigmentary disorders (melasma, Hori's nevus, or acquired pigmentation disorders), as ablative lasers may exacerbate pigmentation and affect study outcomes

### 2.6 Pre‐Treatment Protocol

Patients were instructed to discontinue all topical creams 4 weeks prior to the study, including Vitamin A analogues, bleaching agents, and exfoliants.

The only permitted topical product during this period was sunscreen, which patients were required to use consistently.

The study group, which was randomized to the peeling protocol, followed a two‐week peel regimen beginning 15 days before laser resurfacing, with one rest day (no application) prior to the procedure.

### 2.7 Treatment Protocol

All patients had topical 23% lidocaine and 7% tetracaine in an anhydrous base applied to the target areas 60 min prior to the procedure. The topical anesthetic cream was removed before ablative CO_2_ therapy. Patients underwent treatment with the fractional eCO_2_ Plus laser system (Lutronic Corporation, Billerica, MA, USA), with all procedures performed by a single dermatologist to ensure consistency. Each cosmetic area was initially treated with a single pass, followed by a second pass after all areas had been treated.

### 2.8 Laser Parameters

- Fluences of 110–120 millijoules
- Density of coverage: 5% per pass, for a total of 15% coverage over 3 passes
- Spot size: 120 μm (standardized)

The study area was restricted to the right and left cheeks for standardization in PIHASI assessments. Though some patients received treatment on additional areas (e.g., temples, forehead, nose), these were excluded from the analysis. No epidermal cooling was applied in any cases.

### 2.9 Post‐Treatment Care

Patients in the peel group were restarted on the peel 7 days post‐resurfacing once complete re‐epithelialization was confirmed. They were advised to continue for 4 weeks.

#### 2.9.1 Both Groups Received Standardized Post‐Laser Care, Which Included

- Cetaphil Gentle Skin Cleanser (Galderma, Fort Worth, TX, USA) and white soft paraffin, applied three times daily for 4 days, then twice daily for 3 days.
- Sunscreen (physical‐based SPF 50+), applied twice daily upon re‐epithelialization, regardless of sun exposure. The amount was standardized to two finger lengths per application.
- Study group subjects were instructed to use only the novel peel, with no additional topical preparations.

#### 2.9.2 Medications

- Doxycycline 50 mg once daily for 7 days was prescribed for all participants.
- Two subjects with a history of cold sores received valacyclovir 500 mg twice daily.

#### 2.9.3 Adverse Events

Two cases of mild to moderate skin irritation, characterized by persistent desquamation, pruritus, and mild discomfort, were observed. One case occurred at Weeks 2–3, and the other at Weeks 3–4. Symptoms resolved following temporary cessation of the peel for 4 days, after which treatment was gradually reintroduced from day five.

No cases of prolonged erythema, persistent irritation, or allergic reactions were reported.

### 2.10 Skin Evaluation

All subjects underwent clinical evaluation at Weeks 3 and 6 post‐treatment.

#### 2.10.1 Blinded Assessment

The primary dermatologist was blinded to treatment allocation and assessed outcomes based on standardized photography using the Clinical Imaging System (Sydney, Australia) [23].

#### 2.10.2 Photography Standardization

**FIGURE 2:**
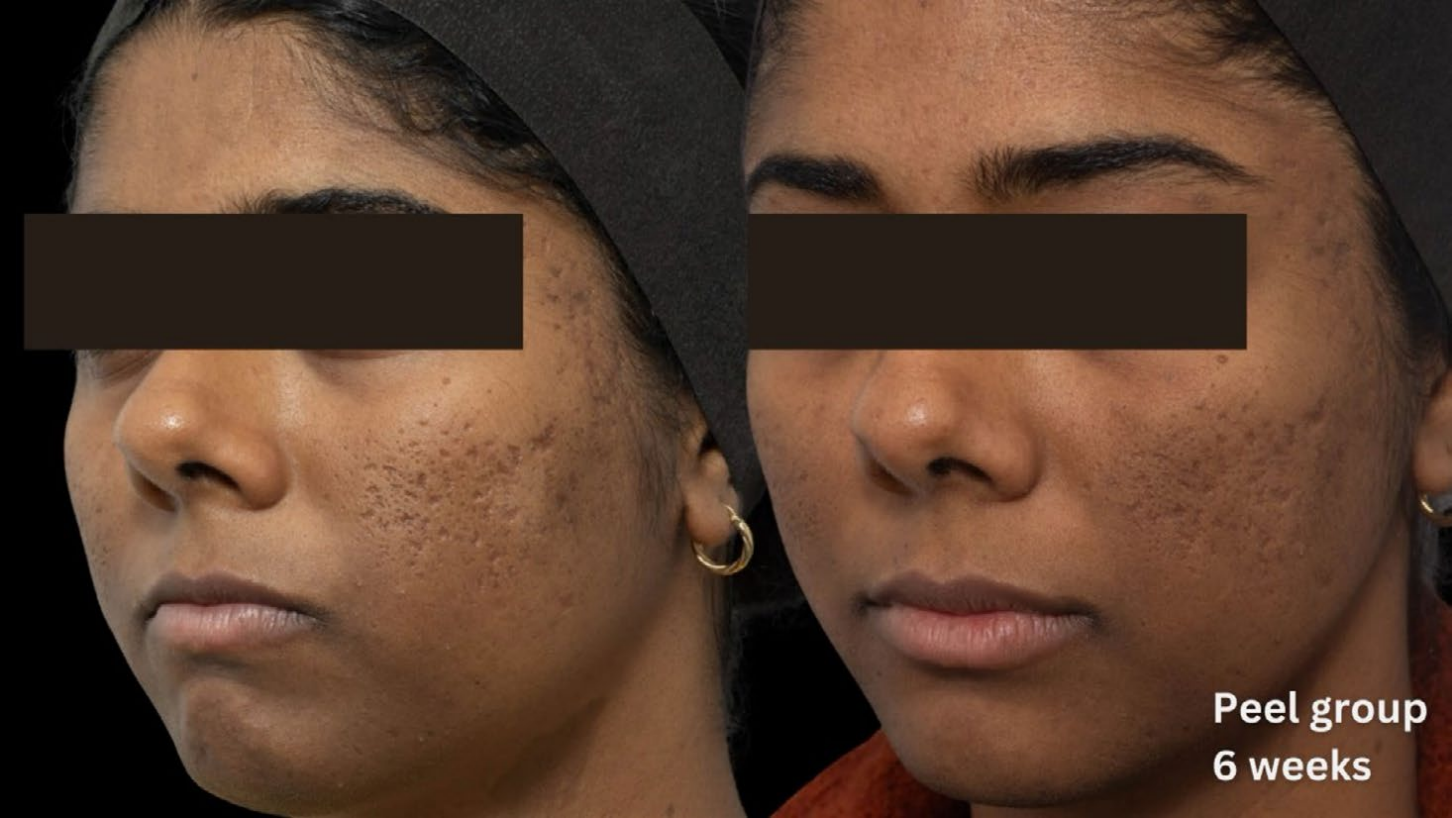
Skin evaluation at 6 weeks for peel group.

**FIGURE 3:**
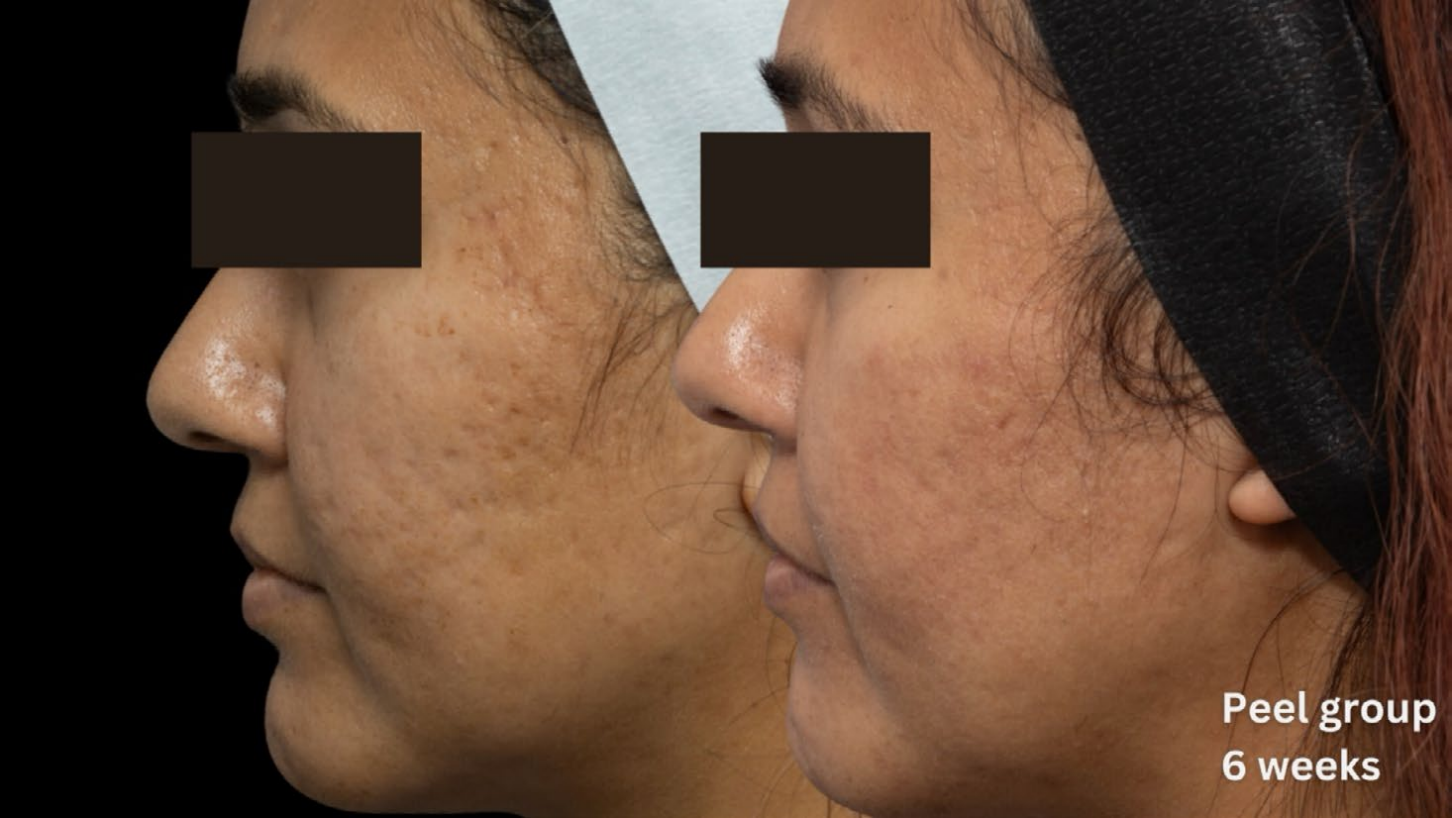
Skin evaluation at 6 weeks for peel group.

**FIGURE 4:**
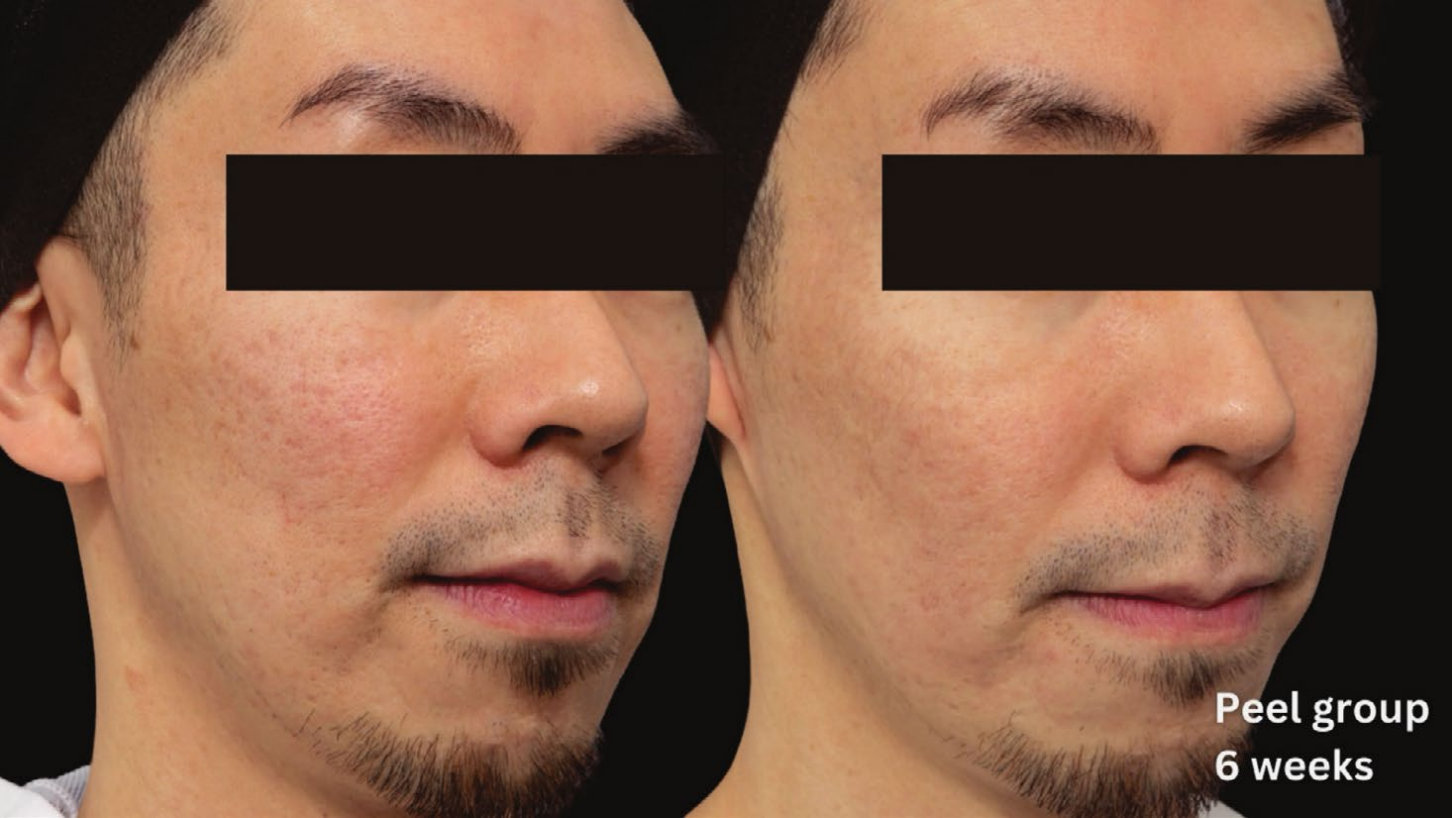
Skin evaluation at 6 weeks for peel group.

**FIGURE 5:**
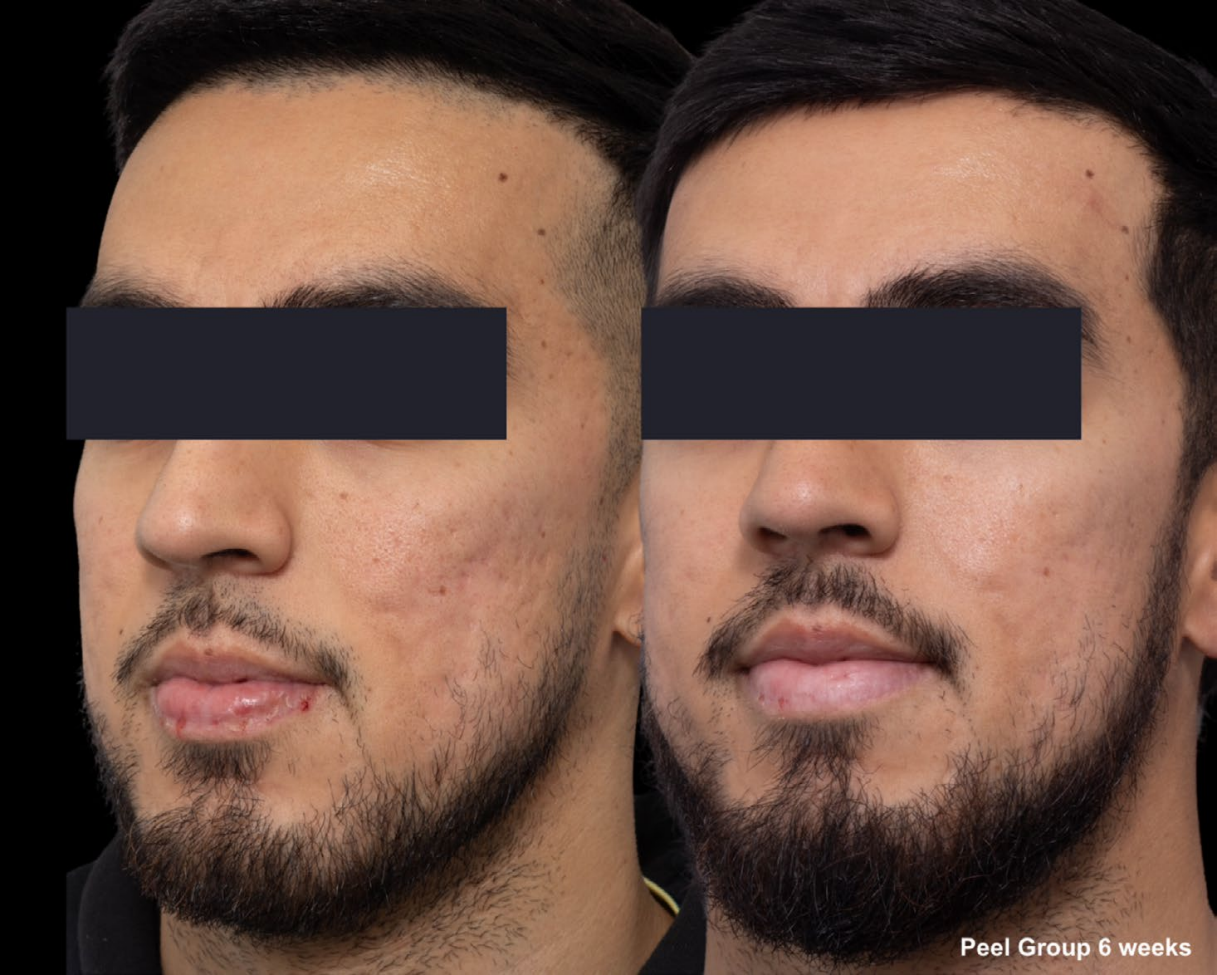
Skin evaluation at 6 weeks for peel group.

**FIGURE 6:**
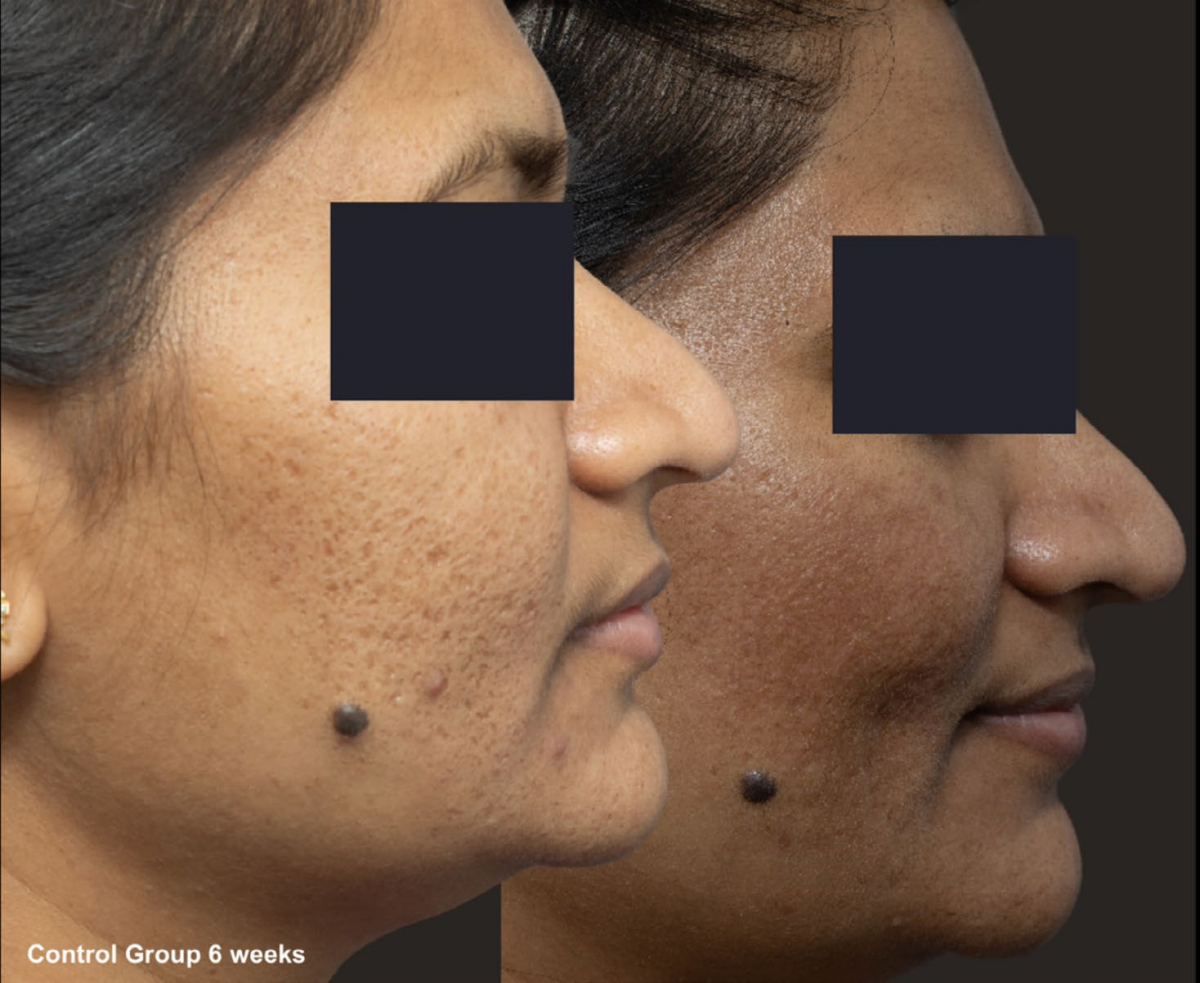
Skin evaluation at 6 weeks for no peel group.

**FIGURE 7:**
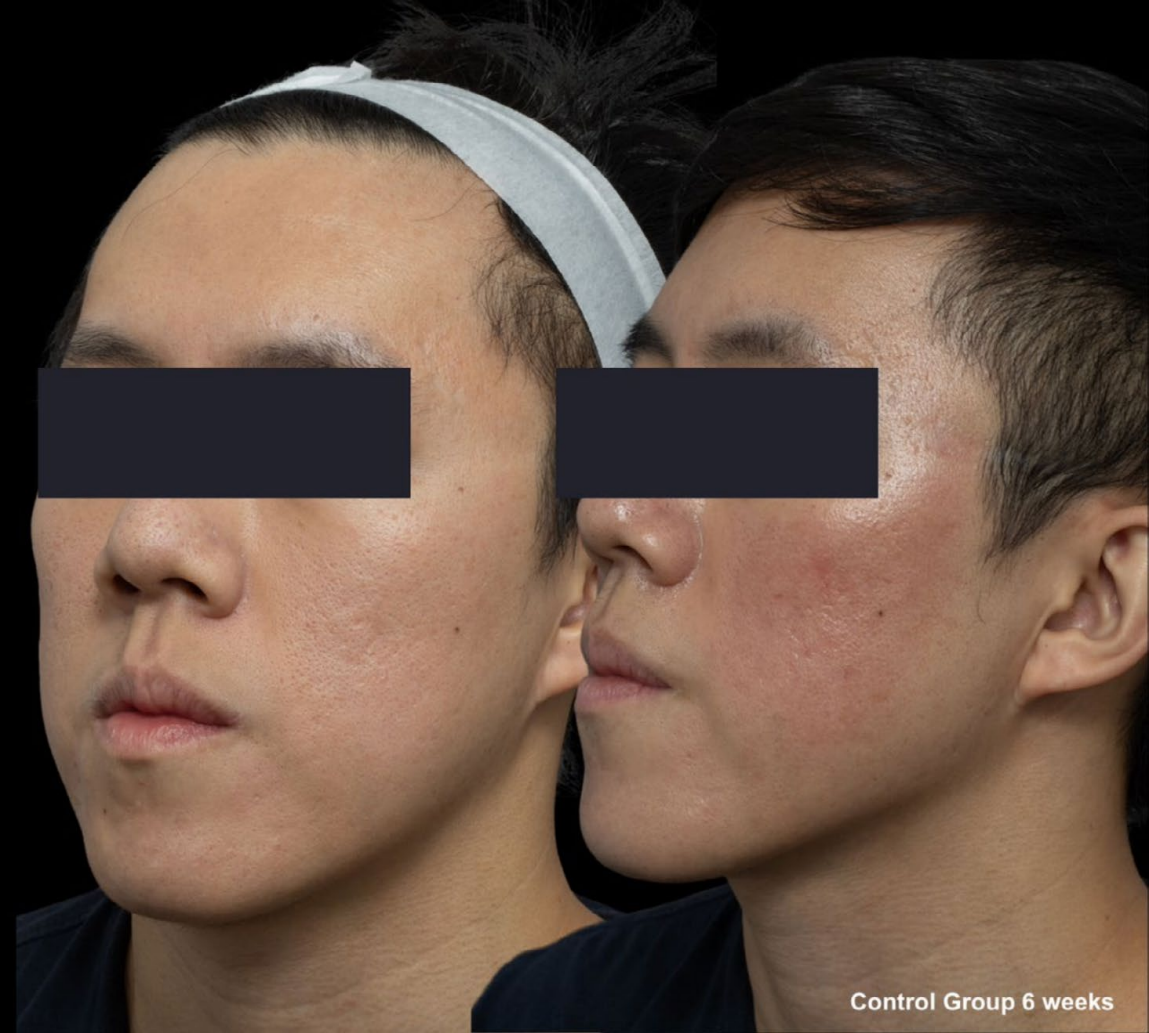
Skin evaluation at 6 weeks for no peel group.

**FIGURE 8:**
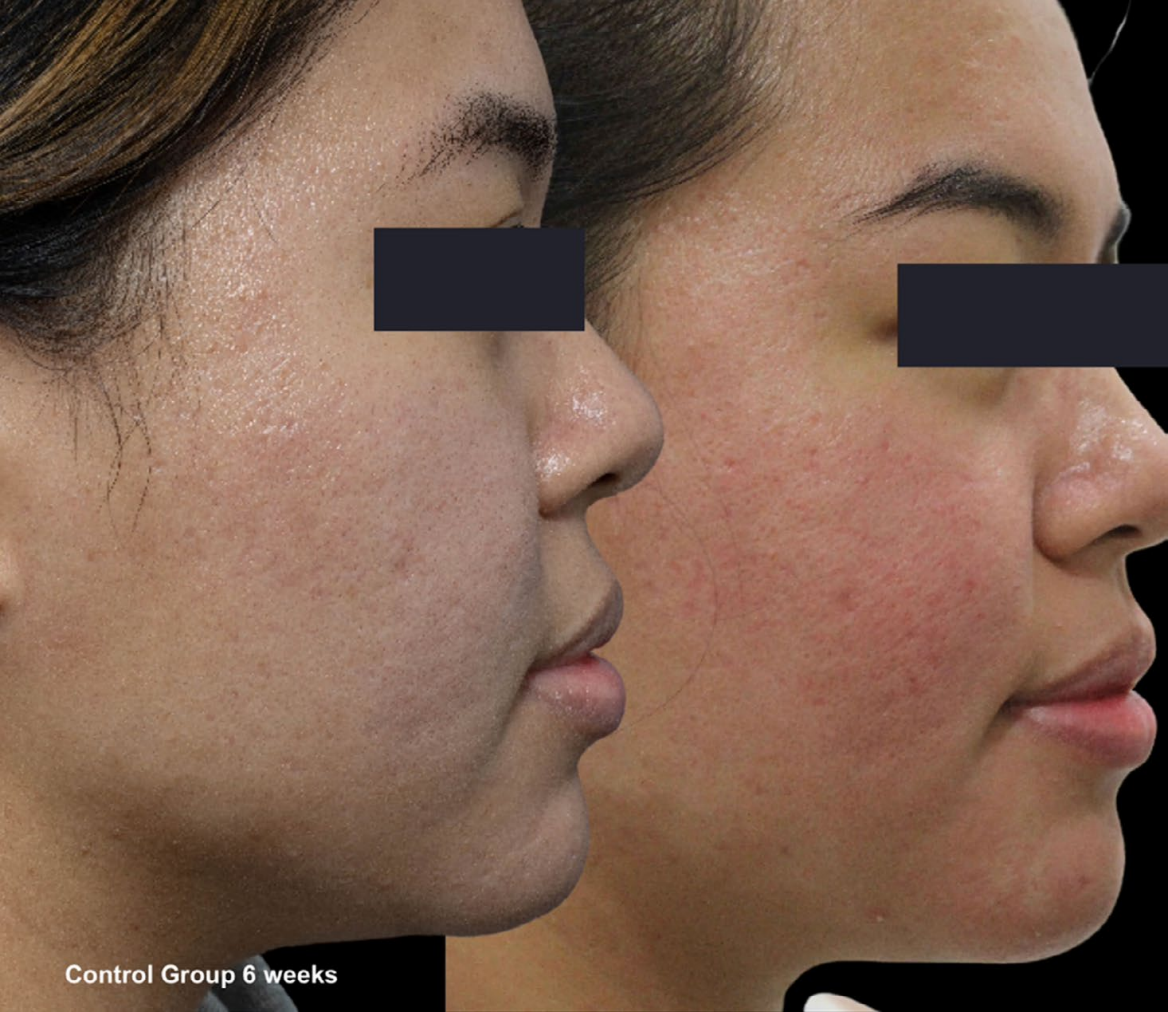
Skin evaluation at 6 weeks for no peel group.

**FIGURE 9:**
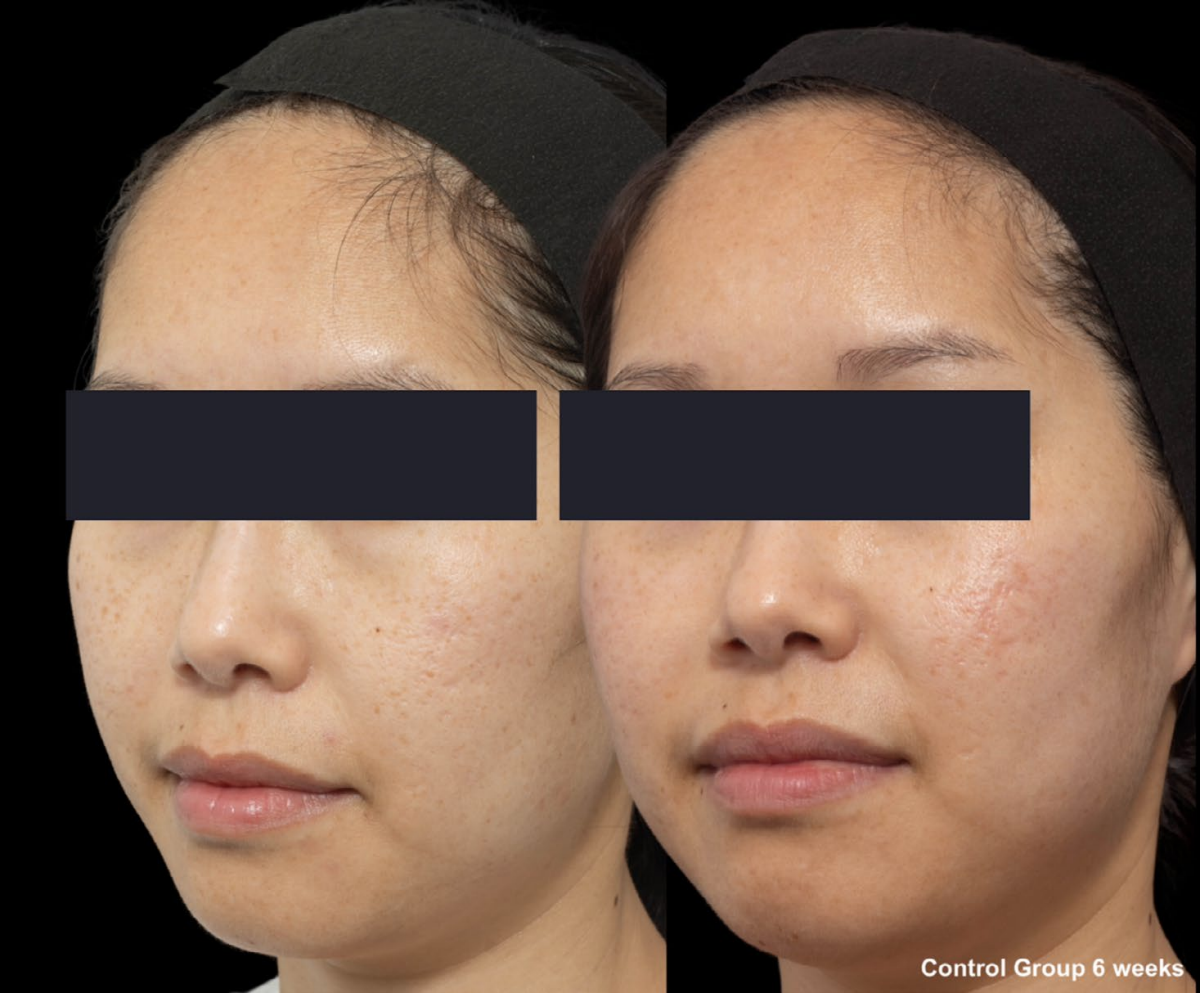
Skin evaluation at 6 weeks for no peel group.

Comparative photographs were taken under controlled conditions to ensure consistent lighting, angle, and exposure. The imaging system was used to maintain fixed distances, lighting, exposure settings, and camera angles, ensuring standardization of photography. Pre‐treatment and 6‐week post‐treatment photographs were captured (Figures 2, 3, 4, 5, 6, 7, 8, 9).

#### 2.10.3 Outcome Assessments

##### 2.10.3.1 Primary Outcome

The primary outcome was the comparison of PIH between the two groups, objectively evaluated at Week 6. The study was terminated at Week 6 post‐procedure, as PIH severity was maximal at Week 4 [11].

PIH is defined as a perceivable increase in pigmentation relative to the surrounding skin. The severity was assessed using the PIHASI score [24]. The PIHASI score was used due to its high interrater reliability, correlation with quantitative hyperpigmentation measures (melanin and Individual Typology Angle values), and concordance with established metrics like the IGA. It provides a broader numerical range, improves the assessment of relative hyperpigmentation, and enhances the specificity of treatment response measurement in inflammatory conditions [25].

PIHASI evaluation included:

- Darkness score (0–4)
- Heterogeneity score (0–4)
- Area percentage (0%–100%)

Severity was determined by summing the darkness and heterogeneity scores. The total score was limited to the face, calculated as 0.1 × F, where F is the product of area and severity.

##### 2.10.3.2 Secondary Outcomes

Healing time (re‐epithelization) and changes in acne scar grading were analyzed using the Goodman and Baron scale. At baseline, patients had grade 4 scars, which were visible at conversational distances and could not be stretched or concealed by normal facial hair growth.

### 2.11 Statistical Analysis

All statistical analyzes were performed using Stata version 18.0.

The primary outcome, PIHASI score at Week 6, was compared between the peel and no‐peel groups using the Mann–Whitney *U* test. Rank sums were used to assess differences between groups, and statistical significance was set at *p* < 0.05.

For secondary outcomes, healing time (re‐epithelialization) and changes in acne scar grading were assessed using mean ± standard deviation (SD) for each group. Differences between groups were analyzed using an independent t‐test (*p* < 0.05).

## 3 Results

### 3.1 Primary Outcome

At 6 weeks post‐treatment, the median PIHASI score in the peel‐treated group was 0.0 (IQR: 0.0–0.2), compared to 0.2 (IQR: 0.2–0.4) in the control group, indicating lower post‐laser pigmentation severity. The difference was statistically significant (*p* = 0.015), supporting the peel's efficacy in reducing PIH risk (Tables 2 and 3).

**TABLE 2:** Peel group PIHASI score at 6 weeks.

Patient number	Darkness (D)	Heterogenicity (H)	Severity (S) = D + H	Area × S	Total score (0.1 × F)
1	1	1	2	1 × 2 = 2	0.1 × 2 = 0.2
2	0	0	0	0	0
3	0	0	0	0	0
4	1	1	2	1 × 2 = 2	0.1 × 2 = 0.2
5	0	0	0	0	0
6	0	0	0	0	0
7	0	0	0	0	0
8	0	0	0	0	0
9	1	1	2	1 × 2 = 2	0.1 × 2 = 0.2
10	2	1	3	1 × 3 = 3	0.1 × 3 = 0.3
11	0	0	0	0	0
12	1	1	2	1 × 2 = 2	0.1 × 2 = 0.2
13	0	0	0	0	0
14	1	1	2	1 × 2 = 2	0.1 × 2 = 0.2
15	0	0	0	0	0

**TABLE 3:** Control (no peel) group PIHASI score at 6 weeks.

Patient number	Darkness (D)	Heterogenicity (H)	Severity (S) = D + H	Area × S	Total score (0.1 × F)
1	1	1	2	1 × 2 = 2	0.1 × 2 = 0.2
2	2	2	4	1 × 4 = 4	0.1 × 4 = 0.4
3	2	2	4	1 × 4 = 4	0.1 × 4 = 0.4
4	2	2	4	1 × 4 = 4	0.1 × 4 = 0.4
5	1	1	2	1 × 2 = 2	0.1 × 2 = 0.2
6	0	0	0	0	0
7	3	2	5	1 × 5 = 5	0.1 × 5 = 0.5
8	1	1	2	1 × 2 = 2	0.1 × 2 = 0.2
9	2	1	3	1 × 3 = 3	0.1 × 3 = 0.3
10	1	1	2	1 × 2 = 2	0.1 × 2 = 0.2
11	0	0	0	0	0
12	1	1	2	1 × 2 = 2	0.1 × 2 = 0.2
13	1	1	2	1 × 2 = 2	0.1 × 2 = 0.2
14	0	0	0	0	0
15	Lost to follow up				

### 3.2 Secondary Outcomes

Re‐epithelialization, defined as the absence of crusting, was similar between groups, occurring at day 6.4 in the peel group and day 6.2 in the control group. Acne scar grading using the Goodman and Baron scores demonstrated no significant difference in both groups (*p* = 0.694), with a mean reduction of 1.27 ± 0.59 in the peel group and 1.36 ± 0.63 in the control group (Table 4).

**TABLE 4:** Characteristics and scar grade changes in the novel peel and control (no peel) groups.

Characteristics	Novel peel group (*n* = 15)	Control group (*n* = 14)
Gender		
Male	4 (27%)	6 (43%)
Female	11 (73%)	8 (57%)
FST		
III	6 (40%)	5 (36%)
IV	7 (47%)	6 (43%)
V	2 (13%)	3 (21%)

	Mean ± standard deviation (range)	Mean ± standard deviation (range)
Scar grade pre‐treatment	4 (All patients)	4 (All patients)
Scar grade post‐treatment	2.73 ± 0.59 (2–4)	2.64 ± 0.63 (1–3)
Scar reduction	1.27 ± 0.59	1.36 ± 0.63
*p* value for scar reduction	0.694	—

These findings suggest that pre‐ and post‐treatment chemical peeling with the MelanoPro Peel System effectively reduces PIH risk following fractional CO_2_ laser resurfacing, particularly in Fitzpatrick III–V patients, who historically face high pigmentation complications. Notably, the 0.0 median PIHASI score in the peel group indicates an absence of visible pigmentation in most treated individuals, highlighting its potential as a standard adjunctive therapy for PIH prevention.

## 4 Discussion

### 4.1 Optimizing CO_2_ Laser Treatment for Acne Scar Remodeling and PIH Prevention

CO_2_ laser resurfacing remains the gold standard for acne scar remodeling, demonstrating efficacy across various scar types in FST I–V.

While not contraindicated in darker skin types (FST IV–VI), its use is challenging due to the high incidence of PIH, reported as high as 100% in some cases [26]. The combination of increased melanocyte reactivity in darker skin and the thermal load of CO_2_ lasers often leads to excessive pigment stimulation and post‐treatment hyperpigmentation.

### 4.2 Prevention and Treatment Strategies for PIH

Given the high risk of PIH, strategic prevention and early intervention are crucial. Various pre‐, intra‐, and post‐procedural strategies have been explored to mitigate pigmentary changes, with strict photoprotection for 2–4 weeks before and after treatment being a long‐standing recommendation. Laser settings are also adjusted to minimize thermal damage, reducing PIH risk [13].

Among topical agents, tyrosinase inhibitors remain the mainstay of PIH prevention, with hydroquinone being the most effective. However, its high irritation potential, safety concerns, and regulatory restrictions limit its universal use [27]. Over the past decade, novel formulations combining multiple tyrosinase inhibitors and exfoliants have emerged as alternatives [26, 28].

### 4.3 The Role of Chemical Peeling in PIH Prevention

Chemical peeling, or chemo‐exfoliation, is a cost‐effective and widely used technique for treating pigmentation disorders. By regulating epidermal thickness, promoting even melanin distribution, and stimulating dermal collagen production, chemical peels improve skin tone and texture. Their efficacy in melasma, acne, PIH, and scarring has been well established, making them a safe and accessible option for pigmentation management [29].

Most studies have focused on post‐treatment PIH management. Our approach, however, emphasizes PIH prevention by integrating a novel peel both as a priming agent before fractional CO_2_ laser resurfacing and post‐treatment intervention to minimize PIH risk. Proper priming is particularly essential in atrophic pigmented acne scars in darker skin types, where high‐fluence laser settings are limited due to PIH risk. Pre‐laser priming enhances skin tolerance, reduces wound healing time, and improves patient compliance [13]. Incorporating antioxidants and anti‐inflammatory cosmeceuticals helps protect against UV damage and prevent further pigmentation [18].

The MelanoPro Peel System is a novel formulation consisting of azelaic acid, tranexamic acid, salicylic acid, glycolic and lactic acids, retinol, niacinamide, and proprietary peptides (Table 5).

**TABLE 5:** Active ingredients found in MelanoPro Peel System (Dermalogica Pro) [30].

Compound	Mechanism of action	Concentration
Azelaic acid	Inhibits tyrosinase, reducing melanin production Exfoliator by removing dull skin surface layers Speeds up skin cell turnover for a brighter complexion	10%
Tranexamic acid (TA)	An amino acid derivative that inhibits plasminogen pathway in melanin synthesis Helps reduce hyperpigmentation and promotes a more even skin tone	2%
Salicylic acid (SA)	Lipophilic, allowing penetration of the epidermal lipid barrier Disrupts corneocyte adhesion, loosening skin cells for exfoliation Promotes dead cell shedding	0.1%
Glycolic (GA)/Lactic acid (LA)	Penetrate the stratum corneum, breaking down bonds between keratinocytes Encourage exfoliation of outer skin layers Provide hydration alongside their exfoliating effects	3.5%/2.7%
Retinol (Vitamin A derivative)	Penetrates the stratum corneum and converts to Retinoic Acid Enhances cell turnover, allowing faster renewal of skin cells Inhibits tyrosinase enzyme involved in melanin synthesis, reducing pigmentation and evening skin tone	0.25%–0.3%
Niacinamide (Pro‐Vitamin B3)	Prevents melanosome transfer to keratinocytes Stopping pigment from reaching the skin's surface	5%
Acetyl Hexapeptide‐1 (Peptide)	Acts as a depigmenting agent, reducing tyrosinase enzyme activity in melanin production Decreases the number, size, and transfer of melanosomes to keratinocytes	—

### 4.4 Azelaic Acid and Its Role in PIH Treatment

Azelaic acid is one of the key ingredients in the MelanoPro Peel System at a concentration of 10%. It was initially formulated as a 20% cream for acne and later as a 15% gel for rosacea. It is now used for PIH due to its ability to inhibit tyrosinase, a key enzyme in melanin production [31]. Additionally, it selectively targets hyperactive melanocytes by inhibiting DNA synthesis and mitochondrial enzymes, leading to cytotoxic effects on abnormal pigment‐producing cells while sparing normal skin. Studies show that a 20% azelaic acid cream significantly reduces pigmentation, and when combined with 15%–20% glycolic acid, its efficacy in treating facial hyperpigmentation, including melasma and PIH, is comparable to 4% hydroquinone [32, 33].

### 4.5 Role of Tranexamic Acid (TA) in Hyperpigmentation Prevention

TA has been shown to effectively modulate melanogenesis by reducing melanin content and tyrosinase activity in melanocytes. It also downregulates key melanogenic proteins such as tyrosinase‐related protein 1 (TRP‐1) and TRP‐2, leading to a reduction in pigment production [34].

Following laser treatments, TA helps mitigate inflammation‐induced melanogenesis by activating the ERK signaling pathway and suppressing melanocyte‐inducing transcription factor (MITF) expression, which plays a crucial role in melanin synthesis [34].

TA has also demonstrated efficacy in melasma management, where it inhibits UV‐induced plasmin activity, thereby reducing arachidonic acid and prostaglandin levels, which are known to stimulate melanocytes and increase pigmentation [35].

While studies indicate that high‐dose oral TA (1500 mg daily) post‐Q‐switched laser does not significantly prevent PIH, evidence suggests that oral administration may facilitate PIH clearance within 6 weeks. This has led to recommendations for further research into pre‐treatment administration to enhance outcomes [17].

### 4.6 Other Active Ingredients in the MelanoPro Peel System

Salicylic acid (SA) is a beta hydroxy acid (BHA) with chemo‐exfoliant properties. It has been widely studied for its effectiveness in treating PIH, particularly in higher FST, both as a standalone chemical peel (30% SA) and in combination with hydroquinone 4% [36, 37]. Although hydroquinone is often used alongside SA, its long‐term inclusion in treatment regimens remains underexplored, and concerns persist regarding its safety profile and potential adverse effects.

Joshi et al. evaluated serial SA peels (20%–30%) in PIH patients (Fitzpatrick IV–VI), comparing treated and untreated facial halves. While mild clinical improvement was observed, the findings were not statistically significant [38].

Ahn et al. reported a whitening effect with 30% SA peels in acne‐induced PIH, noting significant reductions in erythema, oiliness, dryness, and scaliness. This effect is particularly relevant when selecting chemical peels for darker skin tones [39].

Glycolic acid (GA) and lactic acid (LA) are alpha hydroxy acids (AHAs) that suppress melanin formation by directly inhibiting tyrosinase activity, an effect that is independent of their acidic properties. Their role in treating pigmentary lesions extends beyond enhancing epidermal turnover, as they also actively inhibit melanin production within melanocytes. This dual mechanism makes GA and LA valuable agents for addressing hyperpigmentation and improving skin tone [40].

Grover et al. reported that glycolic acid peels resulted in a significant improvement in PIH after 16 weeks [41]. Similarly, Burns et al. found that individuals with darker skin tones experienced faster and more pronounced improvement with glycolic acid peels compared to the control group [42]. This effect is attributed to the exfoliation of pigmented epidermal cells, followed by re‐epithelialization with cells containing less melanin.

Retinol, a derivative of vitamin A, plays a role in PIH treatment by inhibiting tyrosinase transcription and enhancing epidermal turnover, aiding in melanin removal and dispersion. It can be used alone or in combination with other depigmenting agents. Available in concentrations ranging from 0.01% to 0.1%, its use requires caution, particularly in darker skin types, as higher concentrations of tretinoin may cause irritant dermatitis, potentially worsening PIH [43]. The MelanoPro Peel System contains retinol at a concentration of 0.25%–0.3%, which may explain the transient irritant dermatitis observed in two cases within our study. However, symptoms were mild and self‐limiting.

Niacinamide, a form of Vitamin B3, has been studied for its role in treating pigmentary disorders, primarily by reducing melanosome transfer rather than directly inhibiting tyrosinase activity or melanocyte proliferation. Hakozaki et al. demonstrated that niacinamide decreases melanosome transfer by 35%–68%, likely by disrupting the signaling interaction between keratinocytes and melanocytes. Clinical studies have shown that formulations containing 2%–5% niacinamide effectively lighten melasma and UV‐induced hyperpigmentation [44]. However, further research is needed to establish its safety and efficacy in the management of PIH.

Acetyl hexapeptide‐1 is a peptide known for its depigmenting effects, as it decreases melanin production and melanosome transfer to keratinocytes [30]. While extensively used in anti‐aging skincare products for its ability to reduce static and dynamic wrinkles, there is limited published data on its role in pigmentation disorders [45]. This suggests that its application in improving hyperpigmentation may represent a novel and emerging use that warrants further investigation.

## 5 Conclusion

Although the study sample was small, these findings demonstrate that pre‐ and post‐treatment of facial skin in FST III–V with a novel, home‐based peeling system can significantly reduce the incidence of PIH following fractional CO_2_ laser resurfacing. The novel peel is an effective method for minimizing PIH, with high patient satisfaction, low adverse outcomes, and no requirement for hydroquinone prescriptions. In addition to reduced pigmentation, patients reported improvements in overall skin quality, including enhanced texture and reduced roughness. Its non‐prescriptive ingredient list provides a safer and more accessible alternative for patients seeking pigmentation control post‐laser resurfacing.

## Author Contributions

Dr. Xiaozhun Hang and Dr. Davin Lim contributed equally as co‐first authors. Dr. Davin Lim conceptualized the study, led data collection, and drafted the manuscript. Dr. Xiaozhun Hang contributed to statistical analysis, data interpretation, and manuscript revisions. Both authors reviewed and approved the final manuscript.

## Ethics Statement

This study was conducted in accordance with the Declaration of Helsinki. Written informed consent was obtained from all participants before enrolment. Participant confidentiality was maintained, and all data were anonymized before analysis.

## Conflicts of Interest

The authors declare no conflicts of interest.

## Supporting information


Data S1.

## Data Availability

The data that support the findings of this study are available from the corresponding author upon reasonable request.
