# Correction to “*Novel Peel to Prevent Post‐Inflammatory Hyperpigmentation After CO₂ Resurfacing for Acne Scars”*

**DOI:** 10.1111/jocd.70469

**Published:** 2025-09-19

**Citation**

Hang X , Lim DS . A Novel Peel to Prevent Post‐Inflammatory Hyperpigmentation After CO_2_ Resurfacing for Acne Scars. J Cosmet Dermatol. 2025 Aug;24(8):e70366. 10.1111/jocd.70366. PMID: 40736045; PMCID: PMC12309148.40736045 PMC12309148

1. **Funding Statement (page 1):**
  ○ In PubMed indexing (PMID: 40736045), the acknowledgement appears as: *“This work was partially funded by Dermalogica.”*
  ○ However, in the final PDF version of the article, the statement currently reads: *“This work was supported by Dermalogica.”*
  ○ For accuracy and consistency, we respectfully request that the Funding statement in Page 1 of PDF be aligned to: “This work was partially funded by Dermalogica.”
2. **Terminology:**

We request replacement of the product name *“MelanoPro”* with the neutral descriptor *“novel peel system”* (or *“novel peel”*) throughout the article. This change avoids perceived commercial emphasis while maintaining scientific integrity. The specific locations include:

○ **Page 3** ‐ Section 2.2 (Study Objective)
○ **Page 3** ‐ Section 2.3 (Patient Recruitment)
○ **Page 7** ‐ Results (Secondary Outcomes)
○ **Page 9** ‐ Section 4.3, Section 4.4
○ **Page 10** ‐ Table 5 caption
○ **Page 11** ‐ Section 4.6 subheading title
○ **Page 12** ‐ Section 4.6 discussion

**Rationale for post‐publication request**

At the time of proofing, the Funding statement and product terminology were provided by the publisher, and we did not appreciate the inconsistency until after publication. Once the article was indexed in PubMed, it became apparent that the Funding statement in the PDF did not match the indexing record, and that the use of the proprietary product name could be perceived as overly commercial. We are therefore requesting these corrections now to ensure accuracy, neutrality, and compliance with disclosure standards. These corrections are minor, do not affect the scientific content or conclusions, and are requested solely to ensure clarity and neutrality.

We apologize for this error.

Kind regards,

Dr Xiaozhun Hang on behalf of all authors

