# Supplementary material for: A Novel Peel to Prevent Post‐Inflammatory Hyperpigmentation After CO2 Resurfacing for Acne Scars
Source: J Cosmet Dermatol. 2025 Jul 30;24(8):e70366. doi: 10.1111/jocd.70366 (PMC12309148; doi:10.1111/jocd.70366)
Supplement: Supplementary file 1 — Data S1. [file JOCD-24-e70366-s001.pdf]

Post Inflammatory Hyperpigmentation Area and Severity Score (PIHASI)

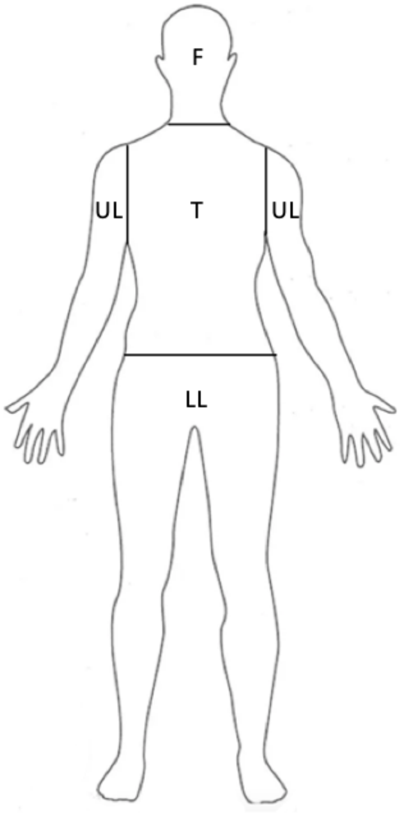

Characteristic	Scoring
Darkness	*0 = absent*<br>*1 = slight*<br>*2 = mild*<br>*3 = moderate*<br>*4 = maximum*
Heterogeneity	*0 = absent*<br>*1 = slight*<br>*2 = mild*<br>*3 = moderate*<br>*4 = maximum*
Area	*0 = 0%*<br>*1 = 1-9%*<br>*2 = 10-29%*<br>*3 = 30-49%*<br>*4 = 50-69%*<br>*5 = 70-89%*<br>*6 = 90-100%*

For each of the four body regions, first calculate severity (S = darkness + heterogeneity):

Region	Face	Upper limbs	Trunk	Lower limbs
**Darkness (D)**				
**Heterogeneity (H)**				
**S = D + H**				

Next, multiply the severity score by area (A) of involvement to yield four sums (F, U, T, L):

Region	Face	Upper limbs	Trunk	Lower limbs
**A * S**	F=	U=	T=	L=

Finally, calculate total PIHASI:

(0.1*F) + (0.2*U) + (0.4*T) + (0.3*L) =

Total PIHASI score ranges from 0-48
